# Genetic diversity of *Rhododendron dauricum* based on morphological traits and SSR markers

**DOI:** 10.3389/fpls.2025.1533824

**Published:** 2025-02-06

**Authors:** Dan Wang, Ying Ma, Xueli Zhao, Ling Wang

**Affiliations:** ^1^ College of Landscape Architecture, Northeast Forestry University, Harbin, China; ^2^ Institute of Forestry, Heilongjiang Academy of Forestry, Harbin, China

**Keywords:** *Rhododendron dauricum* L., phenotypic diversity, genetic diversity, population structure, SSR

## Abstract

*Rhododendron dauricum* L. is one of the most important ornamental plants in Northeast China for its beautiful flowers. Wild *R. dauricum* populations are mainly distributed in Greater and Lesser Khingan Mountains in Heilongjiang, China. The diversity of *R. dauricum* germplasm resources in these areas has not been determined and it can be dynamic due to increasing climate change and human activities, which poses a challenge to effective conservation efforts. To promote genetic diversity conservation and develop new *R. dauricum* varieties, we performed a systematic morphological and molecular evaluation of *R. dauricum* populations in the in 13 populations. The results showed significant inter- and intra-population variations. Clustering revealed 3 major groups. Importantly, plants showing extreme variations in flower color and flower number were used to further develop two new cultivars 'Ao Xue' and 'Yanricai', respectively. In addition, nine SSR markers exhibited polymorphism among the 13 populations, and 25 alleles were found. The Shannon information index (I) was 0.6359, and the polymorphism information index (PIC) was 0.3460. The genetic diversity index (Nei's) was 0.3575, and the observable heterozygosity (Ho, 0.2514) was lower than the expected heterozygosity (He, 0.3722). The average genetic differentiation coefficient (Fst) among populations was 0.6556. Several populations with relatively high genetic diversity including Huzhong, Tahe, and Hongxing were identified. We also found that SSR-based clustering generally follows the geographical distances among the populations. Lastly, we identified two SSR markers that were highly correlated with flower color and leaf aspect ratio. Together, our data provided useful information on the germplasm distribution and variation evolution of *R. dauricum*, which will be valuable for cultivar improvement, protection, and future diversity conservation efforts.

## Introduction

*Rhododendron dauricum* L. is a cold-resistant deciduous semi-evergreen shrub in Northeast China (Ye et al., 2020). *R. dauricum* blooms in early spring and the captivating flower color endows it with high ornamental values. Breeding efforts have made *R. dauricum* popular in landscaping and the cut-flower industry, especially in Heilongjiang, China. *R. dauricum* also showed high medicinal values, making it an important economic plant. Ecologically, *R. dauricum* is the dominant species in the understory shrub layer in the cold temperate coniferous/mixed coniferous forests and thus plays an important role in maintaining ecosystem stability. For example, the dense foliage of *R. dauricum* provides shelter and nesting sites for a diverse array of wildlife. The showy flowers attract pollinators such as bees and insects and thus promote the reproduction of many plant species. The extensive root system can influence the soil conditions by releasing organic compounds and interacting with soil microorganisms (Bai et al., 2011). Given its striking flowers, medicinal uses, and multifaceted ecological roles, there is a growing research interest in *R. dauricum*.

One factor contributing to the aesthetic appeal of *R. dauricum* is the extensive variations in plant morphology. The leaves of *R. dauricum* exhibit a range of shapes and sizes. More importantly, floral diversity can be reflected in many aspects including the petal arrangement, flower size, and coloration, which ranges from pink, purple, to white (Wang et al., 2022). Meanwhile, *R. dauricum* can thrive in many distinct natural habitats including the understory of forests, meadows, and mountainous regions across northeast Aisa. For example, *R. dauricum* grows in a wide latitude and altitude ranges (e.g., 200- 1000 m above sea level). *R. dauricum* can also grow in forests and woodlands along with other trees such as *Larix gmelinii*, *Betula dahurica*, *Quercus mongolica*, *Rosa davurica*, and *Sambucus williamsii*. The ability of *R. dauricum* to colonize diverse habitats underscores its ecological resilience and strong adaptability to different climatic conditions and soil types. It thus raises an interesting question on the putative associations between morphological diversity and the various ecological factors.

There were a few studies examining the genetic diversity of plants in the genus *Rhododendron*. For example, Zhao used AFLP to study seven wild populations of *R. concinnum* in the Qinling area and found that intra-population variation was the major source of genetic diversity (Zhao et al., 2012). Xiao et al. used both SRAP and ISSR markers to analyze the genetic diversity of different species within the genus *Rhododendron* (Xiao et al., 2015, 2016). The results showed extensive genetic diversity at the species level. Similarly, the genetic diversity of five natural populations of *R. fortunei* in Zhejiang Province, China was assessed using ISSR markers, revealing a greater inter-population variation than intra-population variation (Jin et al., 2006). Additionally, the genetic diversity of several other species in the same genus including *R. latoucheae*, *R. hybridum*, *R. shanii*, *R. schlippenbachii*, *R. sinofalconeri*, and *R. aureum* has been reported (Zhao et al., 2010; Ji et al., 2010; Peng et al., 2010; Wu et al., 2013; Zhang et al., 2012, 2021; Zong et al., 2014). Overall, these studies supported the notion that plants in the genus *Rhododendron* are rich in genetic diversity. However, very few studies has assessed the diversity using morphological traits and molecular markers.

The Greater and Lesser Khingan Mountains are the northern boundary of *R. dauricum* in China, in which *R. dauricum* plays an extremely important role in maintaining the stability of the region's fragile ecosystem. With shrinking natural habitats and human destruction, *R. dauricum* is under great threat and the germplasm diversity is unknown. This calls for an urgent need to study the population diversity of *R. dauricum* for practical conservation efforts. Thus, this study aimed to conduct a comprehensive survey of the diversity of *R. dauricum* in the Greater and Lesser Khingan Mountains. We measured various phenotypical traits in 13 representative populations and collected samples for SSR analysis. With detailed variance analysis, we found traits showing the greatest dispersion and populations exhibiting the highest level of variations.

## Materials and methods

### Plant material and sampling

Data on the distribution of *R. dauricum* in seven forestry bureaus in the Greater Khingan Mountains was retrieved (**Supplementary Table S1**). Plants from the following 13 locations were selected for phenotyping and molecular analysis (**Supplementary Figure S1**, **Supplementary Table S2**): Daling (DL), Hongwei (HW), Jiagedaqi (JGDQ), Tahe (TH), Huzhong (HZ), Taoshan (TS), Jinshantun (JST), Hongxing (HX), Meixi (MX), Youhao (YH), Mordauga (MEDG), Haolibao (HLB), and Delbuer (DEBE). They covered the Greater Khingan (DL, HW, JGDJ, TH, and HZ), the Lesser Khingan (TS, JST, HX, MX, and YH), and Inner Mongolia regions (MEDG, HLB, and DEBE) that were representative of the natural habitats of *R. dauricum*. For each population, 30 sampling blocks (5 m x 5 m for each) with an inter-block space of 30 m were set. Data were collected on individual plants of 10-12 years. Sampling was performed on May, 2022.

### Morphological characterization

**Figure 1:**
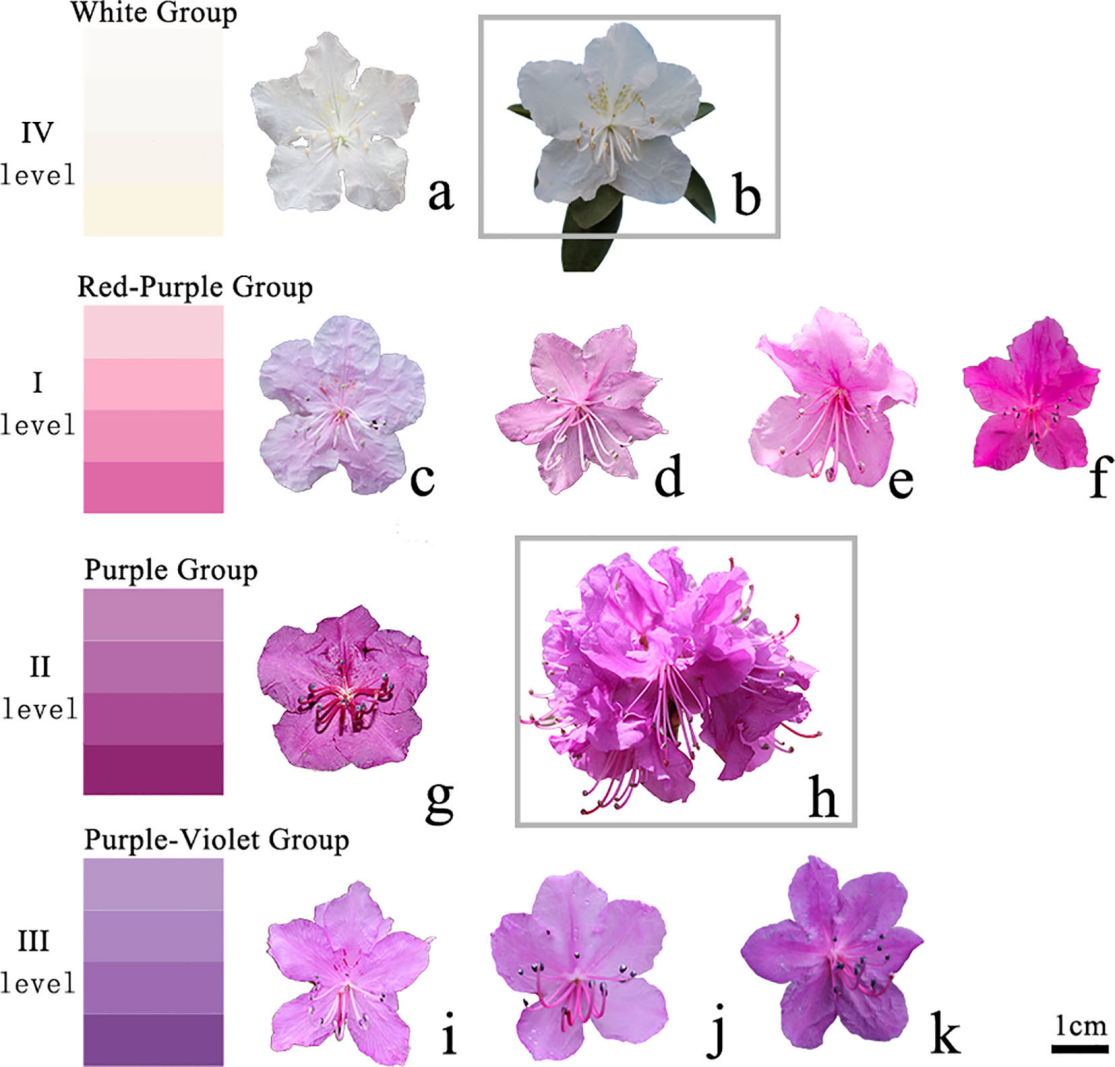
Polymorphism of flower color. IV level **(A, B)**. White group; I level **(C-F)**. Red purple group; II level **(G, H)**. Purple group; III level **(I-K)**. Purple blue group; **(B)** A new cultivar 'Ao xue'; **(H)** A new cultivar 'Yanricai'.

A digital caliper or metric tape was used to measure plant height, ground diameter, and various leaf/flower parameters. Three randomly selected branches were used to measure the ground diameter. Leaves from the current-year branches were measured according to the DUS guidelines developed for *R. dauricum*. Three flowers from each plant were selected to measure the flower diameter. Color determination was performed according to the standards of the Royal Horticultural Society. Specifically, scores 1-3 were assigned to plants with the following flower groups (**Figure 1**): the red-purple (level I), purple (level II), and purple-violet (level III) groups, respectively. In addition, a score of 4 was assigned to white variants (level IV).

We used nine traits for our survey: flower color, flower amount, flower diameter, plant height, ground diameter, branch number, leaf length, leaf width, and leaf aspect ratio. Flower traits were selected because they are the key features determining the ornamental value of *R. dauricum*. Other traits were selected due to their importance to the overall plant shape and the commercial potential of *R. dauricum*.

### SSR markers and genotyping

Dormant branches were sampled in February, 2022 and then stored at room temperature to break dormancy. Flower buds were removed, and the branches were hydro-cultured. Newly emerged leaves (3 to 4 cm long) were used for DNA extraction. Thirty samples were collected for each of the 13 populations. DNA extraction was conducted using the CTAB method (Xiao et al., 2007) with 0.5 g of leaves per sample.

A total of 68 SSR markers were obtained from species closely related to *R. dauricum* (Yang et al., 2018; Zhang et al., 2016; Zhou et al., 2019). These primers were first tested in two plants randomly selected from the 13 populations. Out of these 68 primers (the primer information is shown in **Supplementary Table S3**), 13 showed bright and repeatable bands with polymorphism and were used for further analysis of the 390 samples (30 samples per population; 13 populations). Information on the available primer sequence, length, repeat motifs, and annealing temperature were summarized in **Supplementary Table S4**. For PCR amplification, reactions were performed in 20 μL consisting of 5X PCR buffer, forward primer, reverse primer, DNA template, PCR Enhancer, probe, and H_2_O. The reactions were carried out with the following steps: pre-denaturation at 95°C for 15 min, followed by 35 cycles of amplification (95°C: 30 s, 56°C: 30 s, and 72°C: 30 s); and final extension at 72°C for 3 min. After amplification, electrophoresis detection was performed using the ABI3130xl gene sequencer.

### Statistical analysis

Morphological data was processed with Excel, Spass, and NTSYS (Rohlf, 2000). The degree of dispersion was measured using the coefficient of variation (CV). A two-factor nested design variance analysis was performed to determine the inter- and intra-population variations (Li et al., 2002). The unweighted paired arithmetic mean (UPGMA) method was used for clustering analysis based on the phenotypical data. The neighbor-joining (NJ) method with pair-wise Euclidean distance was used to construct a dendrogram. Associations between phenotypical data and ecological factors including longitude, latitude, altitude, temperature, and precipitation were determined by calculating the Pearson's Correlation coefficient.

The data matrix of the SSR experiment was used to calculate various parameters to infer genetic diversity using Popgene32 and Powermarkersoftware (Liu and Muse, 2005; Yeh et al., 1999). These parameters included number of alleles (Na), number of effective alleles (Ne), expected heterozygosity (He), observed heterozygosity (Ho), Shannon's information index (I), percentage of polymorphic sites (PPL), Nei's gene diversity index (H) (Masatoshi, 1973), gene flow (Nm), gene frequency, site polymorphism information content (PIC) value, genetic differentiation index (Fst). The Chi-square test was used to determine statistical significance. TASSEL was used to determine the correlation between genotype data and phenotypic traits.

## Results

### Overview of the wild populations

**Figure 2:**
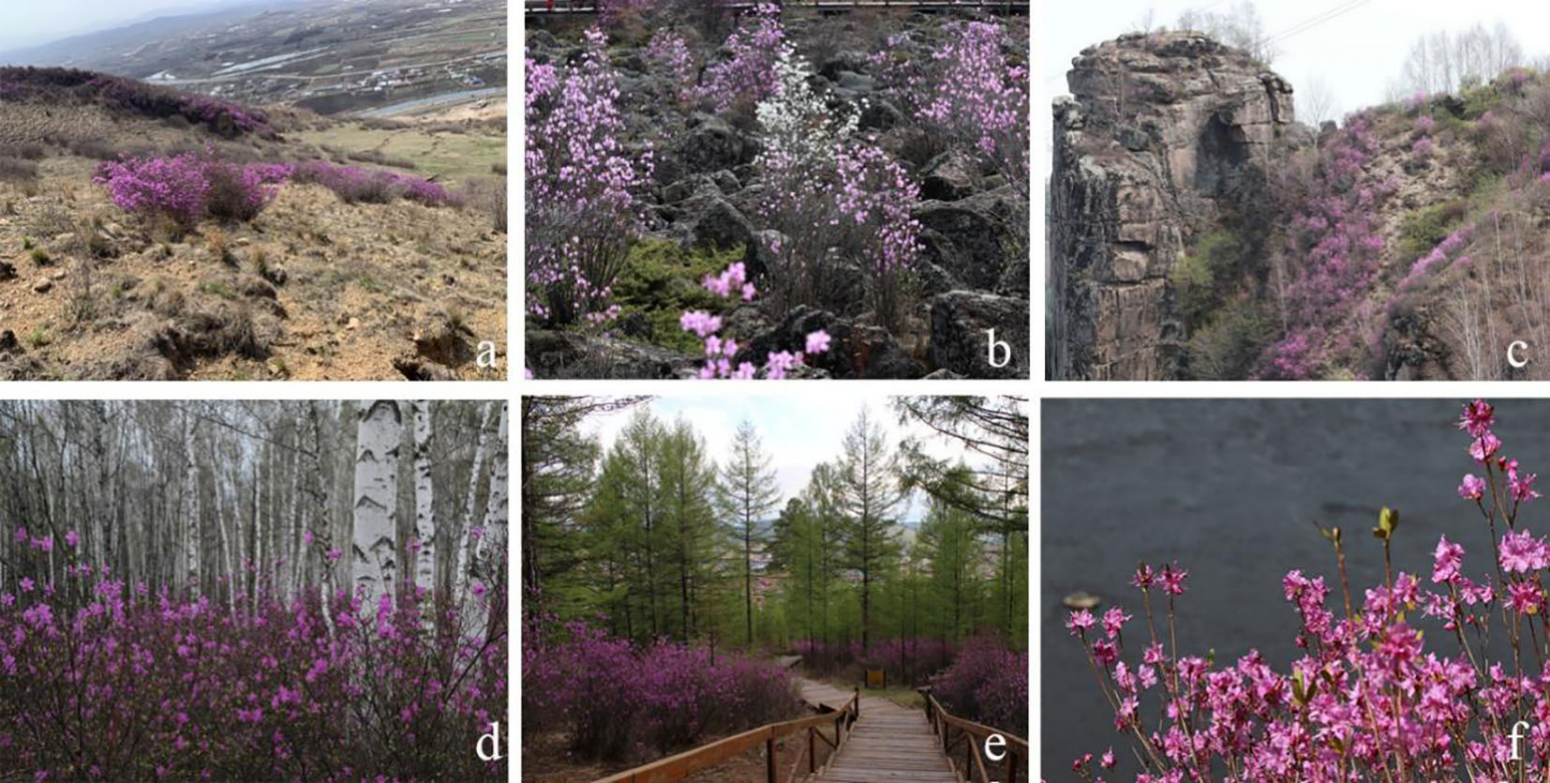
Several representative habitat types of *R. dauricum.* **(A)** Mining site; **(B)** Volcanic rock; **(C)** Sunny slopes; **(D)** Under the *Betula platyphylla* forest; **(E)** Under the *Larix gmelinii* forest; **(F)** River bank.

We first surveyed the distribution of *R. dauricum* in seven forestry bureaus in the Greater Khingan Mountains (**Supplementary Table S1**). A total area of 860,994 hectares of *R. dauricum* was recorded across the seven forestry bureaus. Wild populations of *R. dauricum* are distributed within 122°18'28''E to125°18'37''E and 50°12'21''N to 53°24'17''N, which spans the middle temperate zone and cold temperate zone. *R. dauricum* can grow in multiple habitats including mining sites, volcanic rocks, sunny slopes, forests (*Betula platyphylla* and *Larix gmelinii*), and riverbanks (**Figure 2**). The altitude range is 200 m to 1300 m, although 400 m to 800 m is the preferred altitude. *R. dauricum* thrives in full sun but can tolerate shade. It was usually found in patches with very few individual plants scattered.

A great degree of variation in flowers was observed in the natural populations of *R. dauricum*. First, the corolla was usually shallowly lobed, but deeply or completely lobed corolla was also observed. Five-lobed corollas were the most common ones, while six- or seven-lobed were rare. The flower diameter was 2.5 to 3.5 cm with some reaching up to 4 cm. The number of flowers on a single branch ranged from 1-4. Based on the geographic locations, *R. dauricum* can bloom between late April and early May (Early), from May 1 to May 10 (Middle), and after middle May (Late). In addition, a secondary bloom may occur after the end of September (Secondary).

The flower color varied from white, light purple, pink-purple, purple-red, and purple (**Figure 1**). Interestingly, we found a white variety, which was distinct from *R. dauricum* in terms of corolla color, anther color, filament color, branch color, spot color, and leaf color. This variety had been named *R. dauricum* var. Album (Wilson, 1923). In addition, we found yellow-green spots on some of the white-flowered *R. dauricum*, which had been used to develop 'Ao Xue' via tissue culture and propagation (Wang et al., 2022). Another variety we found showed spherical inflorescences with 8-14 flowers on the same branch, which were significantly more than that in a typical *R. dauricum*. These plants were used to develop a new variety 'Yanricai' (Wang et al., 2023), which had been registered to the Royal Horticultural Society. The numerous flowers make it a variety of high ornamental value.

### Variance Analysis of Phenotypic Traits

To determine the phenotypic trait diversity of the 13 populations, we measured nine traits and summarized their mean values (**Table 1**) and coefficient of variations (CVs, **Table 2**). Based on the inter-population variation, the nine traits were ranked in the order of flower color > flower number > ground diameter > leaf width > branch number > leaf length > leaf aspect ratio > flower diameter > plant height. Of particular interest to us were flower color and number, which showed CVs of 0.2651 and 0.1974, respectively, indicating a large degree of heterogeneity among the populations. It is noteworthy that variations existed for traits showing a small degree of CVs. For instance, the plant height ranged from 158.64 cm (HZ) to 185 cm (MEDG) despite a low inter-population CV (0.0688). We also found that the overall inter-population CV was population-dependent, with HZ and HX showing the largest degree of variations (CVs of 0.1838 and 0.1799 respectively).

**Table 1:** The mean of phenotypical traits and Coefficients of variation (CV) of 13 *R. dauricum* populations.

population	height(cm)/CV	ground diameter(cm)/CV	number of branches/CV	flower diameter(mm)/CV	flower number /CV	flower color/CV	leaf length(mm)/CV	leaf width(mm)/CV	leaf aspect ratio/CV	population CV mean
DL	177.88/0.08	2.75/0.16	20.30/0.1591	37.11/0.0878	406.52/0.1594	2.10/0.3095	34.51/0.1794	13.53/0.2557	2.64/0.1894	0.1756
HW	174.33/0.0271	2.28/0.2281	17.24/0.1868	34.66/0.086	373.39/0.207	1.94/0.2938	33.73/0.1435	15.33/0.1271	2.22/0.1328	0.1591
JGDQ	174.26/0.0261	2.43/0.1975	16.22/0.1967	33.07/0.0765	311.20/0.2714	2.02/0.3069	32.76/0.1361	14.26/0.0998	2.30/0.0861	0.1552
TH	174.76/0.0267	2.23/0.2332	18.03/0.1592	34.89/0.0871	395.79/0.1901	1.87/0.1818	33.75/0.1627	15.21/0.2294	2.25/0.1807	0.1612
HZ	158.64/0.0981	2.54/0.2441	21.46/0.2367	43.19/0.107	479.10/0.173	2.18/0.2982	32.74/0.1426	12.76/0.2053	2.62/0.1489	0.1838
TS	163.88/0.0942	1.93/0.1192	14.10/0.0752	37.83/0.0423	300.50/0.1113	2.17/0.1705	32.45/0.1414	14.45/0.1639	2.26/0.0897	0.112
JST	165.35/0.1263	1.93/0.114	14.71/0.1951	37.71/0.0941	307.39/0.07	2.10/0.1286	32.40/0.1386	14.76/0.1717	2.20/0.0642	0.1225
HX	169.46/0.1426	2.78/0.2338	20.58/0.1433	37.95/0.0991	416.92/0.2361	2.19/0.3059	33.64/0.1356	12.90/0.1752	2.64/0.1477	0.1799
MX	168.19/0.028	1.91/0.1309	14.02/0.174	34.66/0.0906	334.38/0.2415	2.17/0.3041	31.84/0.1856	14.07/0.2658	2.35/0.1957	0.1796
YH	169.07/0.0324	1.92/0.2344	14.27/0.1934	35.24/0.0837	321.93/0.2531	1.99/0.3266	31.58/0.1273	13.85/0.2491	2.37/0.1055	0.1784
HLB	185.06/0.0756	2.48/0.1976	18.62/0.1649	32.59/0.0654	385.10/0.1734	2.13/0.338	31.04/0.0831	13.81/0.1354	2.28/0.1272	0.1512
DEBE	174.06/0.0288	2.21/0.181	17.74/0.1787	34.60/0.0919	386.11/0.2463	2.02/0.2772	34.24/0.153	15.96/0.1769	2.40/0.1292	0.1626
MEDG	188.25/0.1088	2.42/0.1612	18.29/0.1443	32.58/0.0577	382.18/0.2342	2.34/0.2051	30.22/0.1535	13.40/0.1724	2.18/0.187	0.1583
Mean traits	0.0688	0.1873	0.1698	0.0822	0.1974	0.2651	0.1448	0.1868	0.1372	0.1599

**Table 2:** Nested ANOVA of phenotypic characteristics between and within populations of *R. dauricum*.

Trait	Mean square (degrees of freedom)			F	Value
	inter-population	intra-population	error	inter-population	intra-population
height	5596.879(12)	539.410(377)	5.143(780)	1088.181**	104.875**
ground diameter	8.488(12)	1.367(377)	0.940(780)	9.026**	1.454**
number of branches	574.655(12)	20.306(377)	5.830(780)	98.570**	3.483**
flower diameter	763.866(12)	16.441(377)	8.949(780)	85.362**	1.837**
flower number	243983.006(12)	18661.303(377)	877.894(780)	277.919**	21.257**
flower color	1.397(12)	1.138(377)	0.026(780)	54.501**	44.368**
leaf length	148.296(12)	33.637(377)	18.243(780)	8.129**	1.844**
leaf width	81.898(12)	8.452(377)	5.907(780)	13.865**	1.431**
leaf aspect ratio	2.552(12)	0.144(377)	0.121(780)	21.027**	1.183**

Numbers in the parenthesis denotes the degree of freedom; ** denotes statistical significance at an alpha value of 0.01.

To further determine the inter- and intra-population variations, we performed the nested design variance analysis and found that all the nine traits showed significant variations at both the inter- and intra-population levels (α = 0.01; **Table 2**). At the inter-population level, the F statistics ranked the nine traits in the order of plant height > flower number > branch number > leaf length > leaf width > flower diameter > flower color > ground diameter > leaf aspect ratio. At the intra-population level, the order of F values from large to small was plant height > flower color > flower number > number of branches > leaf length > flower diameter > ground diameter > leaf width > leaf aspect ratio.

### Associations between phenotypic traits and ecological factors

Correlations between the nine traits and various ecological factors of the sampling site were summarized in **Table 3**. First, several traits including plant height, ground diameter, branch number, and flower number were negatively correlated to longitude, while flower diameter showed a positive correlation with longitude. Second, plant height, ground diameter, branch number, flower number, and leaf length showed significant positive correlations with the latitude, confirming that *R. dauricum* prefers habits of high latitudes. Third, plant height, branch number, and flower number were extremely significantly positively correlated with the altitude, whilst leaf length and flower diameter decreased as the altitude increased. Fourth, the annual average temperature showed a positive impact on several traits (flower diameter and leaf length) and a negative one on others (plant height, ground diameter, branch number, flower color, and flower number). Lastly, the annual precipitation showed a positive correlation with flower diameter and negative correlations with plant height, ground diameter, and branch number.

**Table 3:** Correlation analysis between phenotypic characteristics and ecological factors of *R. dauricum*.

ecological factors	height	ground diameter	number of branches	flower diameter	flower color	flower number	leaf length	leaf width	leaf aspect ratio
longitude	-0.344^**^	-0.136^**^	-0.322^**^	0.222^**^	-0.014	-0.256^**^	0.013	-0.022	0.044
latitude	0.218^**^	0.177^**^	0.439^**^	-0.037	-0.045	0.362^**^	0.086^**^	0.024	0.049
altitude	0.349^**^	1.000	0.205^**^	-0.261^**^	0.000	0.161^**^	-0.066^**^	0.001	-0.044
annual average temperature	-0.161^**^	-0.229^**^	-0.087^**^	-0.113^**^	-0.059^**^	-0.215^**^	-0.070^**^	-0.032^**^	-0.036^**^
annual precipitation	-0.330^**^	-0.343^**^	-0.139^**^	0.220^**^	-0.006	-0.270	-0.025	-0.059^*^	0.053

* and ** denote statistical significant at alpha values of 0.05 and 0.01, respectively.

The greater the genetic distance, the smaller the genetic consistency, which proves that the farther the genetic relationship between the two; on the contrary, when the genetic distance is smaller, the greater the genetic consistency, the higher the genetic relationship. It can be seen from the **Table 4** that the genetic distance between HX and DL was 0.0417, the genetic identity was 0.9592, and the genetic relationship between them was the closest. The genetic distance and genetic identity between JGDQ and MEDG were 0.4463 and 0.64, and the genetic relationship between them was the farthest.

**Table 4:** Analysis of genetic distance and genetic consistency.

popID	MX	DEBE	HX	YH	JGDQ	HZ	TH	HW	TS	MEDG	JST	HLB	DL
MX	****	0.8333	0.8087	0.8909	0.8573	0.8909	0.8686	0.7833	0.8333	0.7348	0.8333	0.9167	0.8573
DEBE	0.1823	****	0.7661	0.8018	0.7757	0.8018	0.8909	0.8704	0.7917	0.7348	0.7917	0.7917	0.7757
HX	0.2123	0.2664	****	0.637	0.8549	0.7963	0.7963	0.7557	0.8725	0.7507	0.8513	0.7661	0.9592
YH	0.1156	0.2209	0.451	****	0.6983	0.8095	0.8095	0.7444	0.6682	0.6547	0.6682	0.8018	0.6983
JGDQ	0.1539	0.254	0.1568	0.3591	****	0.851	0.8292	0.8102	0.8981	0.64	0.8573	0.7757	0.88
HZ	0.1156	0.2209	0.2278	0.2113	0.1613	****	0.881	0.884	0.8241	0.6547	0.7572	0.8018	0.7856
TH	0.1409	0.1156	0.2278	0.2113	0.1873	0.1268	****	0.9305	0.8463	0.7856	0.8018	0.8463	0.7856
HW	0.2442	0.1388	0.2801	0.2952	0.2105	0.1233	0.0721	****	0.8269	0.6822	0.7398	0.7398	0.7249
TS	0.1823	0.2336	0.1363	0.4032	0.1074	0.1935	0.1668	0.1901	****	0.694	0.9583	0.7917	0.8573
MEDG	0.3081	0.3081	0.2868	0.4236	0.4463	0.4236	0.2413	0.3824	0.3653	****	0.694	0.8573	0.72
JST	0.1823	0.2336	0.161	0.4032	0.1539	0.2781	0.2209	0.3013	0.0426	0.3653	****	0.7917	0.8573
HLB	0.087	0.2336	0.2664	0.2209	0.254	0.2209	0.1668	0.3013	0.2336	0.1539	0.2336	****	0.7757
DL	0.1539	0.254	0.0417	0.3591	0.1278	0.2413	0.2413	0.3217	0.1539	0.3285	0.1539	0.254	****

The upper triangular matrix is genetic consistency, and the lower triangular matrix is genetic distance.

****Indicates that the genetic distance is 0 and the genetic consistency is 1.

**Figure 3:**
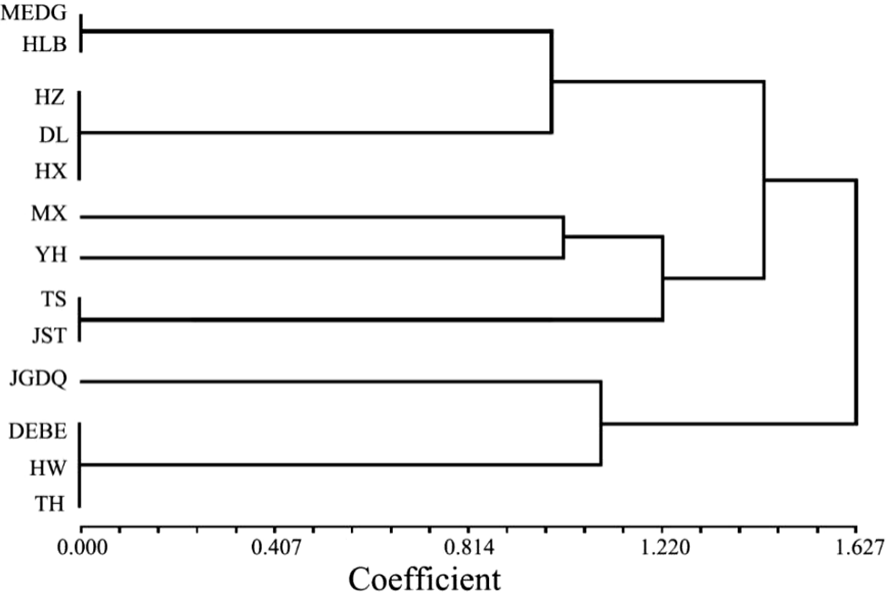
UPGMA cluster based on the phenotypic characteristics of 13 populations in *R. dauricum*. Sample codes in this image represent the same *R. dauricum* samples as **Supplementary Figure S1**.

The UPGMA dendrogram constructed by similarity coefficient data obviously showed that three clusters were formed when the similarity coefficient was 1.30 (**Figure 3**). The resulting dendrogram revealed three major groups. The first group consisted of MEDG, HLB, HZ, DL, and HX. The second group comprised MX, YH, TS, and JST. The third group included JGDQ, DEBE, HW, and TH. The group 1 accessions were mainly *R. dauricum* collected from the western Greater Khingan Mountains, HX accessions were also assigned into this groups. All four Lesser Khingan Mountains accessions were classed to group 2. The *R. dauricum* genotypes of group 3 were primarily collected from the east of the Greater Khingan Mountains. While most populations clustered according to geographical distance, there were exceptions. This indicated a discontinuity in the phenotypic trait variation of the 13 populations.

### SSR markers

Cross-species microsatellite markers are powerful for studying the genetic diversity of species whose genome information is unavailable. We randomly selected 68 pairs of published SSR primers from the same genus and found that the majority resulted in positive PCR bands (41 out of 68). This supports the conserveness in the flanking sequences of SSR among closely related species (Guo, 2007; Nie, 2013; Wang et al., 2010a, 2016, 2010b). Based on the band clarity, we selected 13 SSR markers to further evaluate the genetic diversity of 390 individuals from the 13 populations (**Table 5**). Four markers including Rd-SSR-43, Rd-SSR-60, Rd-SSR-9, and Rd-SSR-65 resulted in a single band, indicating monomorphism of the locus. Nine pairs of SSR primers (Rd-SSR-53, Rd-SSR-34, Rd-SSR-51, Rd-SSR-38, Rd-SSR-59, Rd-SSR-57, Rd-SSR-16, Rd-SSR-66, and Rd-SSR-19) showed multiple bands and thus polymorphism among the 13 populations.

**Table 5:** Genetic diversity of 9 SSR marker in 13 *R. dauricum* populations.

Locus	Sample Size	Major.Allele. Frquency	Na	Ne	Obs_Het	Exp_Het	Nei**	Ave_Het	I*	PIC	HWE-P
Rd-SSR-53	26	0.6923	3.0000	1.8571	0.3846	0.48	0.4615	0.1923	0.7903	0.4042	ns
Rd-SSR-19	24	0.9231	1.0000	1.0000	0	0	0	0	0.0000	0.1319	ns
Rd-SSR-34	24	0.8077	2.0000	1.2800	0.25	0.2283	0.2188	0.1154	0.3768	0.3032	ns
Rd-SSR-51	26	0.8462	2.0000	1.3520	0.1538	0.2708	0.2604	0.0769	0.4293	0.2265	ns
Rd-SSR-38	26	0.7692	2.0000	1.5505	0.4615	0.3692	0.355	0.2308	0.5402	0.2920	ns
Rd-SSR-59	26	0.6538	2.0000	1.8270	0.5385	0.4708	0.4527	0.2692	0.6450	0.3502	ns
Rd-SSR-57	24	0.3846	7.0000	4.0563	0.1667	0.7862	0.7535	0.0769	1.6410	0.7605	**
Rd-SSR-16	26	0.9615	2.0000	1.0799	0.0769	0.0769	0.074	0.0385	0.1630	0.0712	ns
Rd-SSR-66	26	0.4615	4.0000	2.7934	0.2308	0.6677	0.642	0.1154	1.1374	0.5742	**
Mean	25.33	0.7222	2.7778	1.8662	0.2514	0.3722	0.3575	0.1239	0.6359	0.3460	--
St.Dev	0.9428	0.1856	1.6850	0.9270	0.1683	0.2442	0.2344	0.0846	0.4752	0.2021	--

ns is not significant; **, extremely significant (alpha = 0.01); *, significant (alpha = 0.05).

The nine pairs of polymorphic primers covered 25 alleles, with 1-7 alleles per marker. The number of effective alleles ranged from 1 to 4.0563 (mean of 1.8662). The Shannon information index I ranged from 0 to 1.6410 (mean of 0.4752). Polymorphism information index (PIC) ranged from 0.0712 to 0.7605, identifying Rd-SSR-57 and Rd-SSR-66 as the preferred markers for genetic diversity studies in *R. dauricum*. The observable heterozygosity (Ho) ranged from 0 to 0.5385 (mean = 0.2514) and the expected heterozygosity (He) ranged from 0 to 0.7862 (mean = 0.3722), suggesting the presence of inbreeding and genetic variation among the populations. This was further confirmed by the average heterozygosity (Ave_Het), which ranged from 0 to 0.2692. In addition, the genetic diversity index Nei ranged from 0 to 0.7535 (mean = 0.3575) and indicated that the genetic diversity varied greatly among the alleles.

We also determined the genetic diversity among the 13 *R. dauricum* populations (**Table 6**). The average number of alleles is about 15 with HZ and TH populations showing 18 alleles. There was a general agreement between the observed heterozygosity and the expected heterozygosity, indicating random breeding of these populations. JGDQ and DL showed the lowest observed heterozygosity, and HZ and TH showed the largest. Accordingly, HZ and TH showed the highest Nei's gene diversity index values (0.1923 for both).

**Table 6:** Genetic diversity of 13 *R. dauricum* populations.

Population	Na	Obs _Het	Exp_Het	Ave_Het	Nei's	I	PPL
MX	15	0.1538	0.1538	0.0858	0.0769	0.1066	15.38%
DEBE	16	0.1538	0.1538	0.0858	0.0769	0.1066	15.38%
HX	16	0.2308	0.2308	0.0858	0.1154	0.1600	23.08%
YH	14	0.0909	0.0909	0.0909	0.0455	0.0630	7.69%
JGDQ	14	0.0769	0.0769	0.0858	0.0385	0.0533	7.69%
HZ	18	0.3846	0.3846	0.0858	0.1923	0.2666	38.46%
TH	18	0.3846	0.3846	0.0858	0.1923	0.2666	38.46%
HW	15	1.1667	1.1667	0.0865	0.0833	0.1155	15.38%
TS	15	1.1538	1.1538	0.0858	0.0769	0.1066	15.38%
MEDG	14	0.0769	0.0769	0.0858	0.0385	0.0533	7.69%
JST	14	1.1538	1.1538	0.0858	0.0769	0.1066	15.38%
HLB	15	1.1538	1.1538	0.0858	0.0769	0.1066	15.38%
DL	14	0.0769	0.0769	0.0858	0.0385	0.0533	7.69%
Mean	15.2308	0.4813	0.4813	0.0862	0.0868	0.1204	17.16%

### Spatial genetic structure analysis of populations

**Figure 4:**
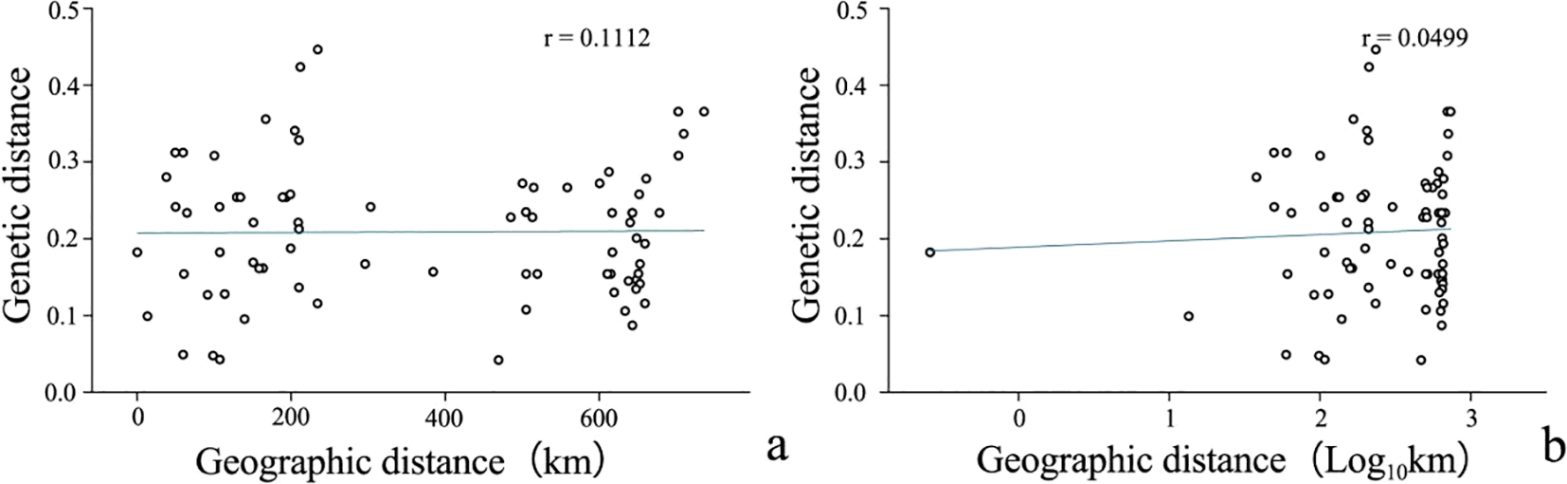
Correlation between genetic distane(log) and geographical distance(log) of *R. dauricum* populations. **(A)** Correlation between genetic distance and geographical distance; **(B)** Correlation between genetic distance log and geographical distance log.

Pearson correlation analysis of genetic and geographic distances was performed using Origin. The results are shown in **Figure 4A** (r=0.1112) and **Figure 4B** (r=0.0499), which shows that there is a certain correlation between genetic distance and geographic distance between populations, but the correlation is not significant. This indicates that genetic variation has little correlation with geographic distance. This may result in lower gene exchange between populations, leading to genetic differentiation between populations.

### Divergence among 13 populations based on SSR analysis

**Figure 5:**
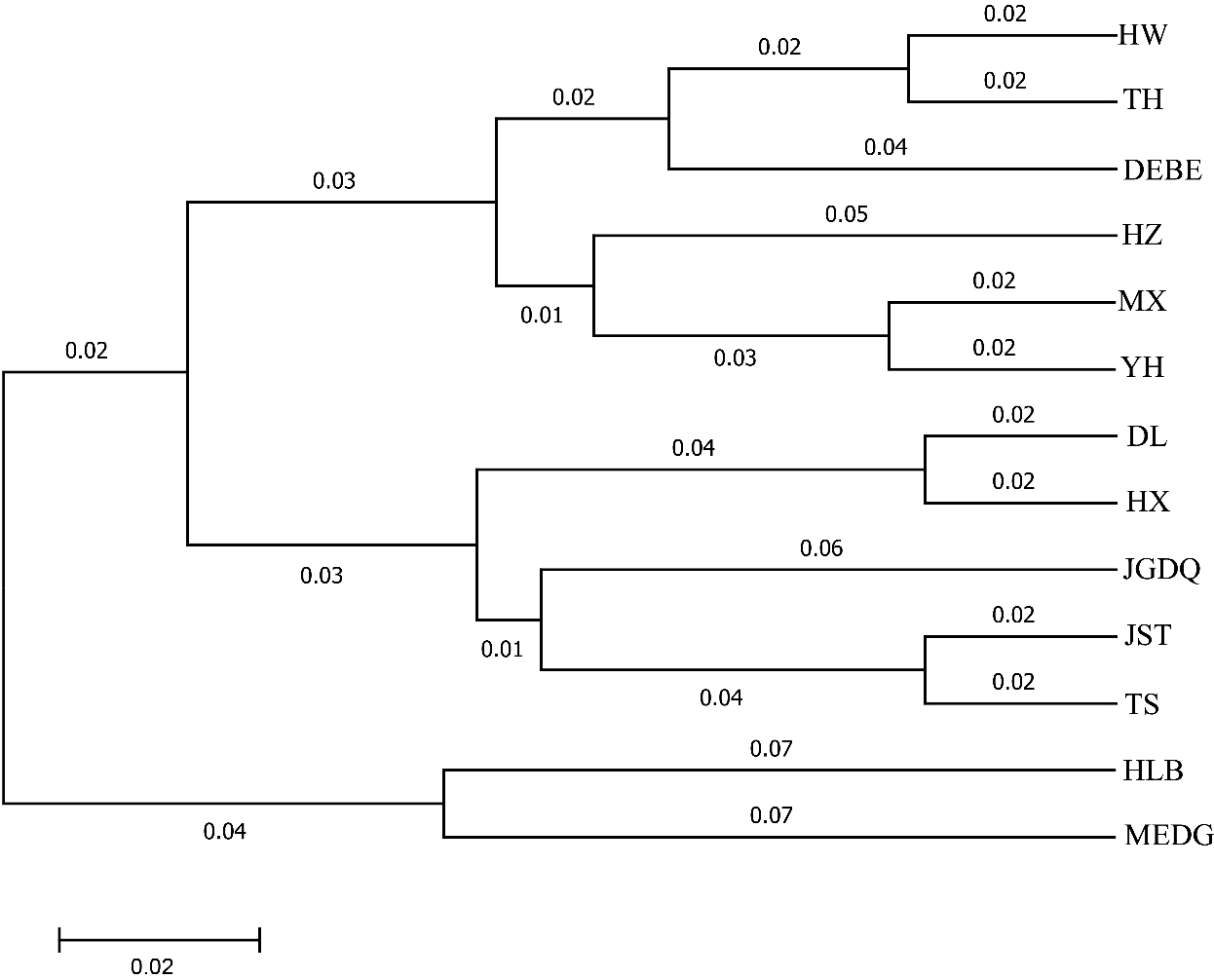
Cluster diagram genetic distance among *R. dauricum* populations.

**Figure 6:**
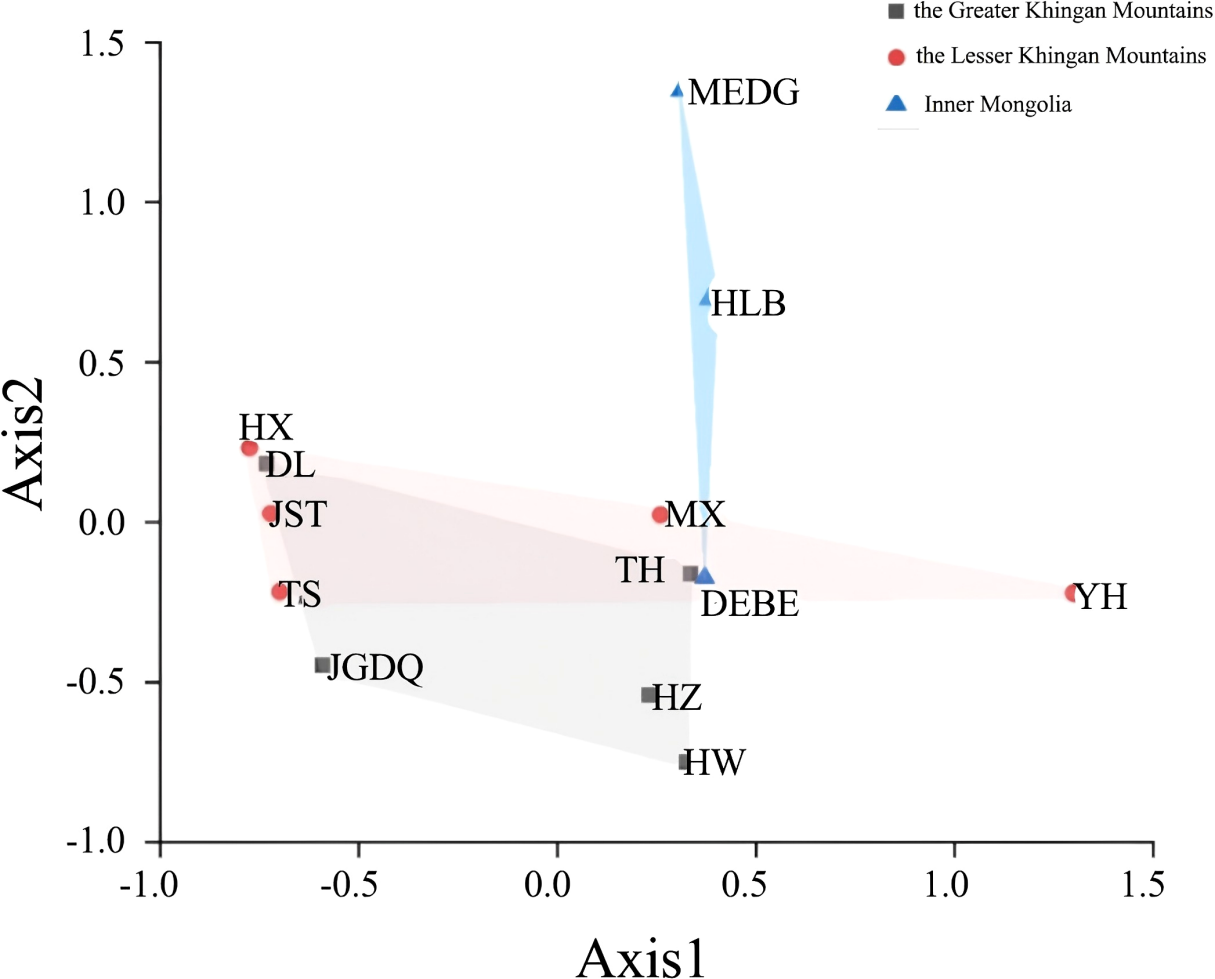
Principal coordinate analysis. Points of different colors and shapes represent sample groups in different environments.

Next, we used Nei's F statistic to analyze the SSR data and found that the Fst values ranged between 0.35 and 1 (**Table 7**), with an average inter-population Fst of 0.6556. Thus, the within-population variance (34.44%) was lower than the between-population variance (65.56%). Clustering analysis revealed three groups in the phylogenetic tree (**Figure 5**, **Supplementary Table S5**) with a clear separation among HLB, MEDG, and the remaining 11 populations. The relatively large genetic distance between HLB/MEDG and other populations can also be reflected in the principal coordinate plot (**Figure 6**). Overall, we observed an increase in genetic distance among the populations as their geographical distance increased, consistent with the geographical distance model (Jiang et al., 2016). Similar genetic backgrounds were found in relatively close populations, for example, between HW and TH, HLB and MEDG, JST and TS, and MX and YH. However, deviation from this model was also common, such as HZ clustered closely with MX although they were geographically distant. Many factors including human activities and insect pollination may be behind this, which requires further investigation.

**Table 7:** ** **F Statistics and gene flow analysis of microsatellite sites.

Locus	Sample	Fis	Fit	Fst	Nm*
Rd-SSR-53	26	-1	0.1667	0.5833	0.1786
Rd-SSR-19	24	****	1.0000	1.0000	0.0000
Rd-SSR-34	24	-1	0.3097	0.6549	0.1318
Rd-SSR-51	26	-1	0.4091	0.7045	0.1048
Rd-SSR-38	26	-1	-0.3000	0.3500	0.4643
Rd-SSR-59	26	-1	-0.1895	0.4052	0.3669
Rd-SSR-57	24	-1	0.8052	0.9026	0.0270
Rd-SSR-16	26	-1	-0.0400	0.4800	0.2708
Rd-SSR-66	26	-1	0.6406	0.8203	0.0548
Mean	26	-1	0.3113	0.6556	0.1777

* Nm = Gene flow estimated from Fst = 0.25(1 - Fst)/Fst.

****Indicates that the degree of inbreeding of the population cannot be judged by this value. This table mainly calculates Nm based on the FST value.

### Association between phenotypic traits and SSR markers

**Figure 7:**
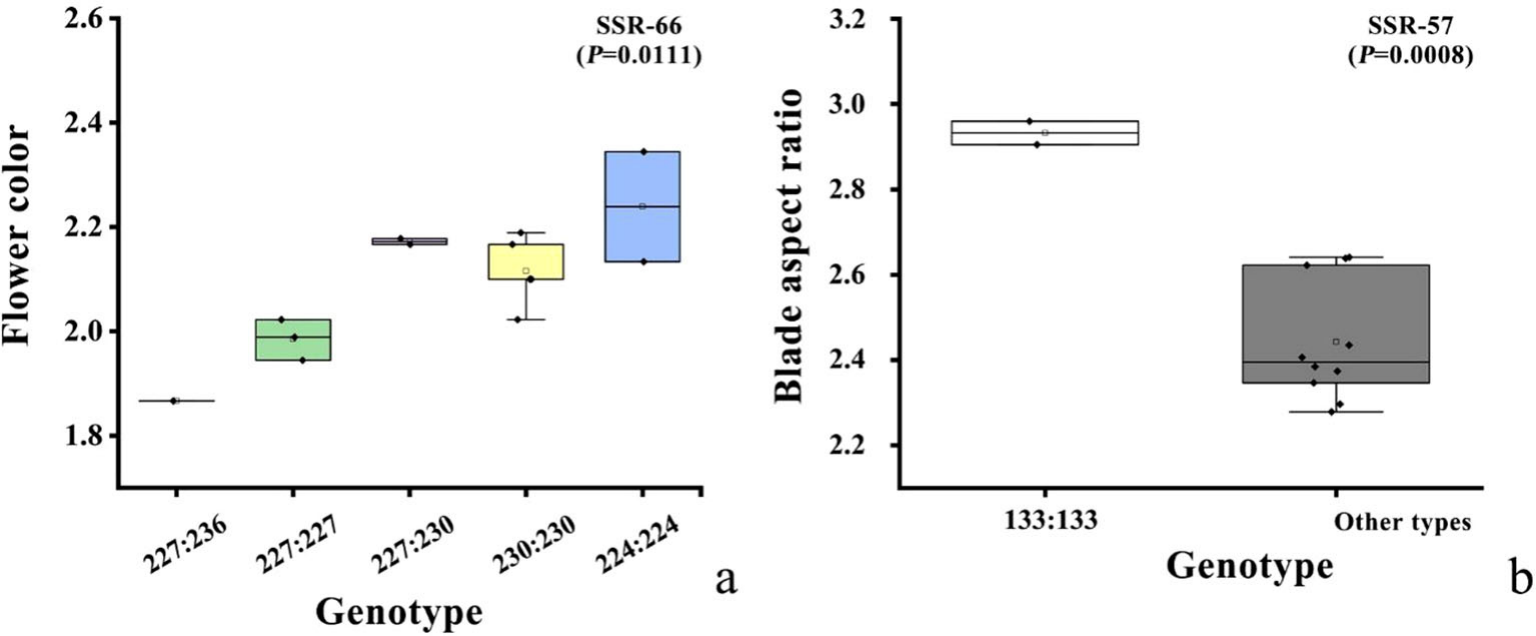
Box diagrams showing the distribution of flower color **(A)** and leaf aspect ratio **(B)** across genetic background.

We next used GLM to determine the association between SSRs and phenotypic traits and found that 3 loci with statistical significance (*p* < 0.05, **Table 8**): Rd-SSR-57 for leaf aspect ratio, ground diameter, branch number, and flower number; Rd-SSR-66 for flower color and plant height; and Rd- SSR-34 for plant height and flower diameter (**Supplementary Figure S2**). The explanation rate of phenotypic variation by individual SSRs ranged from 0.3179 to 0.7110 (**Table 8**). Among them, Rd-SSR-66 showed an explanation rate of 0.7110 for flower color, while both Rd-SSR-66 and Rd-SSR-57 were significantly correlated with flower color and leaf aspect ratio (*Q* < 0.1). Further analysis revealed a significant variance in flower based on Rd-SSR-66 genotypes, with genotypes 244:244, 230:230 and 227:230 showing darker flower color in TH, HW, DEBE, YH and 227:227 and 227:236 corresponding to lighter flower color values in MEDG and HLB (**Figure 7A**). For Rd-SSR-57, a significant difference was found in leaf length-to-width ratio between TS/JST and the remaining populations (*P* =0.0008, **Figure 7B**).

**Table 8:** Trait-marker association analysis based on GLM model.

Trait	SSR	*F* value	*P*	*Q*	*R^2^*
Leaf ratio	Rd-SSR-57	7.9438	0.0103	0.0490	0.3179
Flower color	Rd-SSR-66	6.6690	0.0144	0.0950	0.7110
Plant height Plant height	Rd-SSR-66	6.6449	0.0145	0.1040	0.6412
	Rd-SSR-34	5.7481	0.0246	0.1840	0.5039
Ground diameter	Rd-SSR-57	5.8631	0.0234	0.1370	0.5398
Flower diameter	Rd-SSR-34	4.9644	0.0352	0.3530	0.4354
Branch number	Rd-SSR-57	4.7960	0.0382	0.2200	0.4928
Flower number	Rd-SSR-57	4.5985	0.0421	0.2930	0.5050

## Discussion

In our study, the nine SSR markers covered 25 alleles with a PIC value of 0.3460 and an average Shannon information index I of 0.6359. At the population level, the average expected heterozygosity was 0.4813, and the average percentage of polymorphic sites was 17.16%. These numbers were lower compared to those reported in a previous study by Jiang et al (Jiang et al., 2016). The discrepancy could be attributed to differences in the population location and size in the two studies (**Supplementary Figure S3**). Population size is of particular importance because a small population size will reduce the evolutionary potential of wild species and thus the genetic diversity (Frankham, 1996). In addition, previous research showed that the genetic background of *R. dauricum* populations in the Greater and Lesser Khingan Mountains is similar (Yang et al., 2020) and they are all derived from Siberian flora. There was no expansion in the wild habitats of *R. dauricum* after the Ice Age (Jiang et al., 2016). Thus, genetic exchange among different populations was unlikely, causing isolated refugia. In supporting of this, our data demonstrated that the genetic imprints from different refuges still profoundly affect the population structure of *R. dauricum* in Northeast China.

Our data indicated a great inter-population variation with a Fst value of 0.6556. This was different from Jiang et al. (Fst = 0.2089). Again, the inconsistency can stem from variations in sampling and the choice of molecular markers. In addition, a low Nm value of 0.1777 from our analysis suggested a low level of gene flow among the populations. This is supported by the fact that *R. dauricum* assumes habitats of distinct geological conditions. Thus, long-term geographical isolation and natural selection promote divergences. Accordingly, our data showed a certain correlation between genetic distance and geographical distance (**Figure 4**), but it is not significant. The same reason could explain the results of the clustering analysis, in which most populations clustered in accordance to geographical distances with some expectations. For example, HW and TH, JST and TS, MX and YH, HLB and MEDG, are all clustered together for geographic proximity, which is consistent with the phenotypic markers(**Figure 5**). Exceptions were indeed observed, For example, HX and DL are not clustered by geographic distance, possibly due to human activities or animal migration.

We found both common and unique patterns in the clustering results based on either phenotypic or molecular data. However, discrepancies were also obvious including the grouping of HZ, DL, and HX in phenotypic clustering and the separation of HZ from DL and HX in clustering results based on SSR data. The inconsistency is not uncommon, as reported in previous studies. It can be caused by several factors. Phenotypic traits are extremely susceptible to external conditions including environmental factors (Zheng et al., 2016). By contrast, molecular markers are more stable but the selected ones in any study may not be strongly associated with the phenotypic traits. In fact, one major limitation of this study is that only 9 SSR markers were used here, which resulted in only a small set of detectable alleles. Regardless of the methodology used, both data in our study showed a high level of genetic diversity among the 13 populations and identified populations that should be prioritized for conservation efforts (e.g.,HZ and HX). In addition, we found a high correlation between flower color, leaf length-to-width ratio, and molecular markers. Thus, the two methods provided complementary information.

Research results indicate that (Lu et al., 2022), due to the ‘self-thinning effect’, the population of *R. dauricum* in Greater Khingan Mountains shows a declining trend, with the number of juvenile individuals decreasing and the number of older individuals increasing over time in the middle and lower elevation birch forests. This suggests that changes in the population of *R. dauricum* are influenced not only by external factors such as human activity but also by internal natural dynamics of the population itself (Zhang et al., 2004).

On one hand, human activities are a major external factor increasing survival pressure and reducing the population size of *R. dauricum*. The plant forms flower buds by late summer, which need to overwinter and bloom the following spring. However, human activity, such as breaking branches in winter, severely impacts flowering and seed production the next year, hindering population reproduction. Additionally, the damaged forest understory resources cannot quickly recover, leading to insufficient reserves of seedlings and young trees, creating significant gaps, and in some cases, even causing resource extinction through excessive harvesting. This severely disrupts the balance of the forest ecosystem and affects population numbers. Furthermore, global warming is contributing to the marshification of *R. dauricum* habitats, which is another external factor leading to population decline.

On the other hand, the internal factors contributing to the vulnerability of *R. dauricum* are primarily influenced by seed reproductive capacity and viability, pollinators, and seed physiological ecology. In wild populations of *R. dauricum*, seedling numbers are low, and some individuals rely on clonal reproduction to maintain population renewal, with seedlings exhibiting slow growth. This could be due to a thick litter layer or competition for resources from other species within the same community, leading to difficulties in seed germination and severe limitations on seedling growth, making population recovery challenging.

Previous studies have shown that *R. dauricum* is self-affection and, in the wild, its main pollination method involves cross-pollination assisted by pollinators, consistent with the reproductive systems of other species within the *Rhododendron* genus (Mejías et al., 2002; Zhang et al., 2021). The survey also revealed that while there are various flower-visiting insect species, the effective pollinators are primarily bumblebees (Fenster et al., 2004; Xiaoling et al., 2011). Additionally, the flowering period is only 7 to 10 days and occurs in the early spring of the northeastern forest region, often experiencing late spring cold snaps. This results in frequent pollination failures and severe reproductive disorders, hindering the population's ability to reproduce and regenerate.

Since our data showed that the within-population variation is the major contributor to the genetic diversity of *R. dauricum*, future conservation effort should prioritize populations with a high level of diversity. Accordingly, priority should be given to HZ, TH, and HX. Human interference should be minimized in these areas. In addition, inter-population mating can be considered to promote gene exchange. With the help of field reintroduction and population reconstruction, the wild population will be gradually regenerated and restored.

## Conclusion

The present study underscores that both phenotypic traits and molecular marker analysis can reflect the high genetic diversity of *R. dauricum* in the Huzhong,Tahe and Hongxing populations. Therefore, priority should be given to protecting these populations. Simultaneously obtaining two latent sites with high significance for leaf aspect ratio and flower color, respectively. It can be used to distinguish differences between populations and molecular marker assisted breeding.

## Data Availability

The original contributions presented in the study are included in the article/**Supplementary Material**, further inquiries can be directed to the corresponding author/s.
