# Supplementary figures and images for: Genetic diversity of Rhododendron dauricum based on morphological traits and SSR markers
Source: Front Plant Sci. 2025 Feb 6;16:1533824. doi: 10.3389/fpls.2025.1533824 (PMC11839661; doi:10.3389/fpls.2025.1533824)

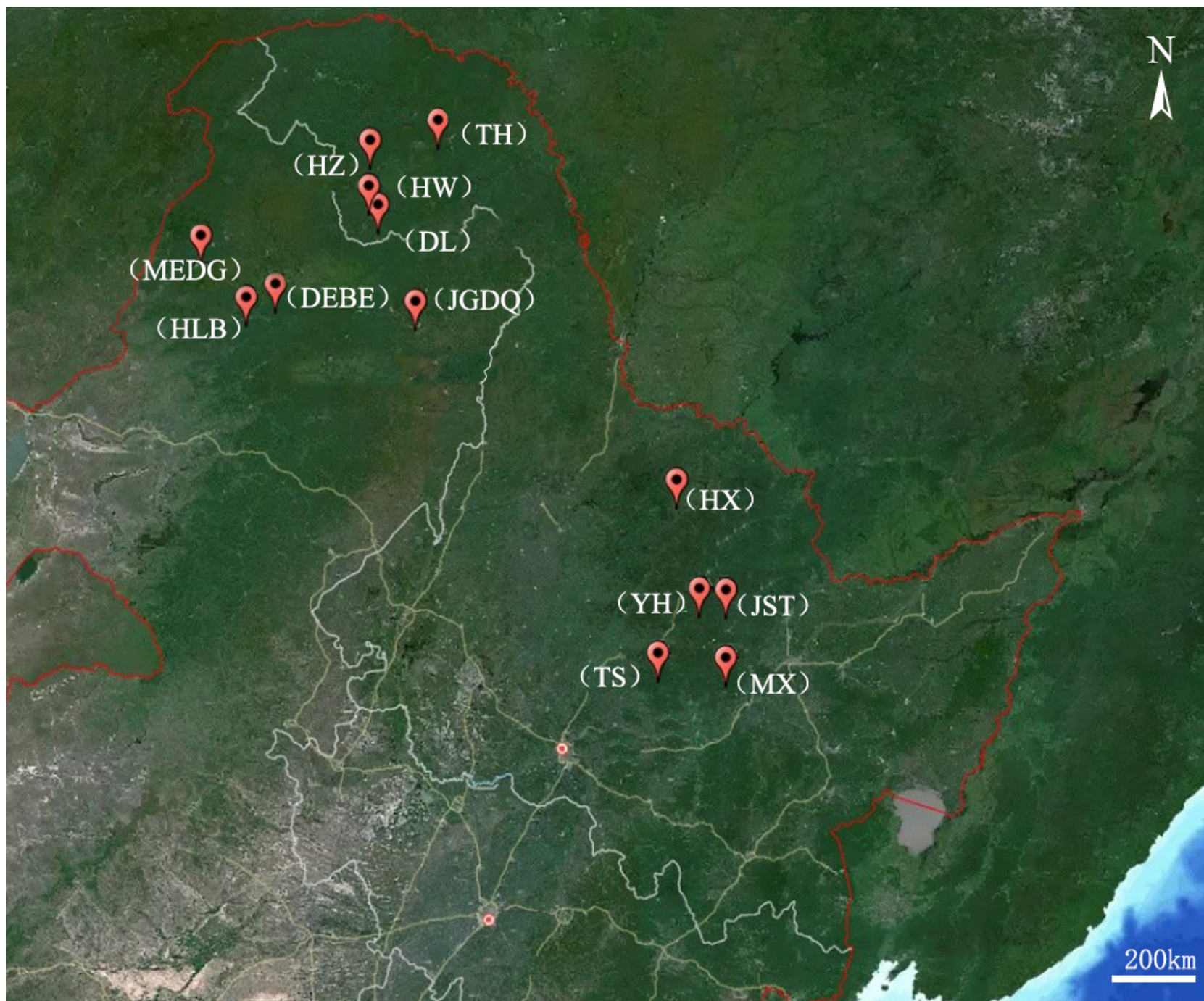

Supplement: Supplementary file 1 [file Image1.pdf]

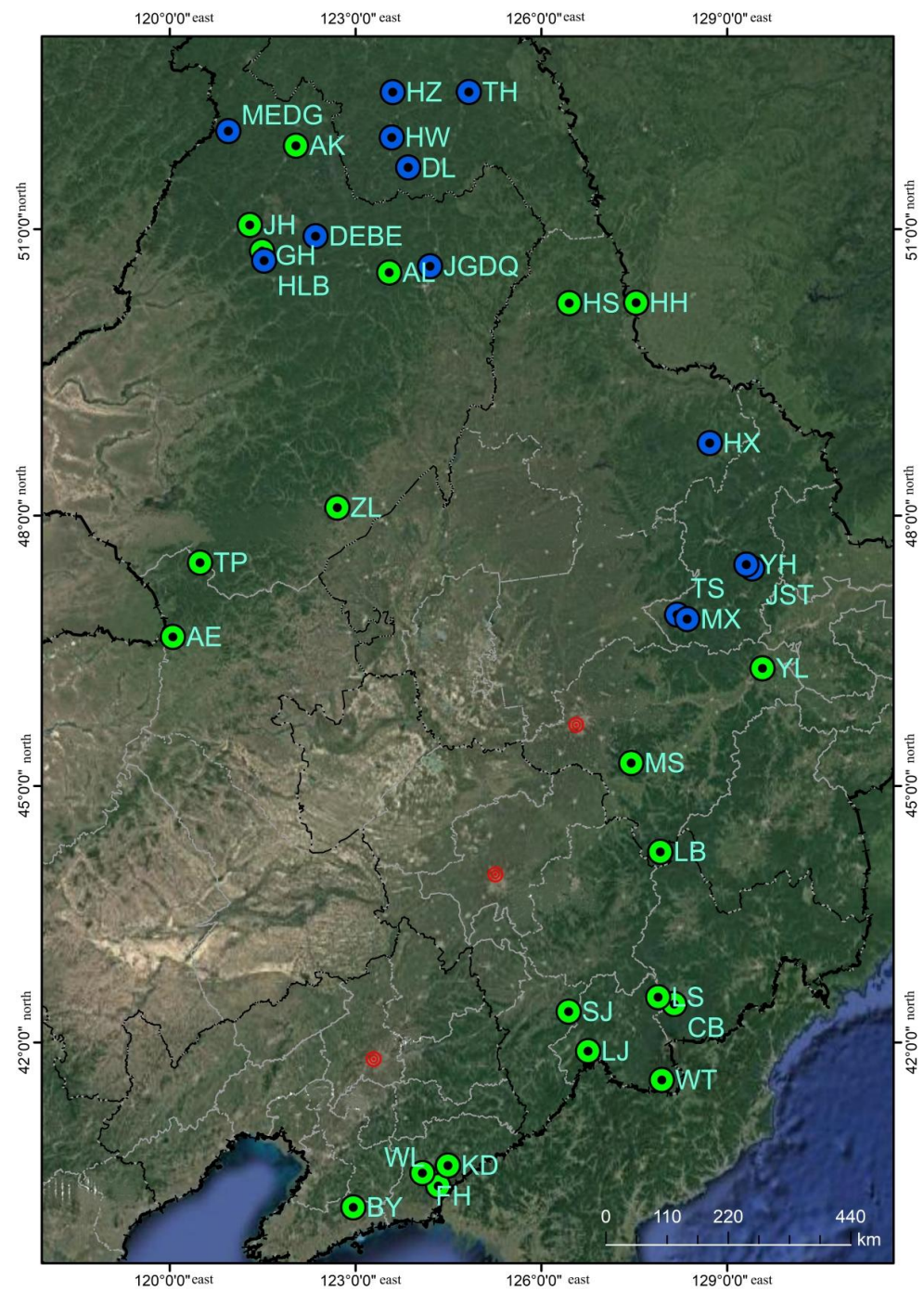

Supplement: Supplementary file 3 [file Image3.pdf]
