# Supplementary material for: Genetic diversity of Rhododendron dauricum based on morphological traits and SSR markers
Source: Front Plant Sci. 2025 Feb 6;16:1533824. doi: 10.3389/fpls.2025.1533824 (PMC11839661; doi:10.3389/fpls.2025.1533824)
Supplement: Supplementary file 2 [file Image2.pdf]

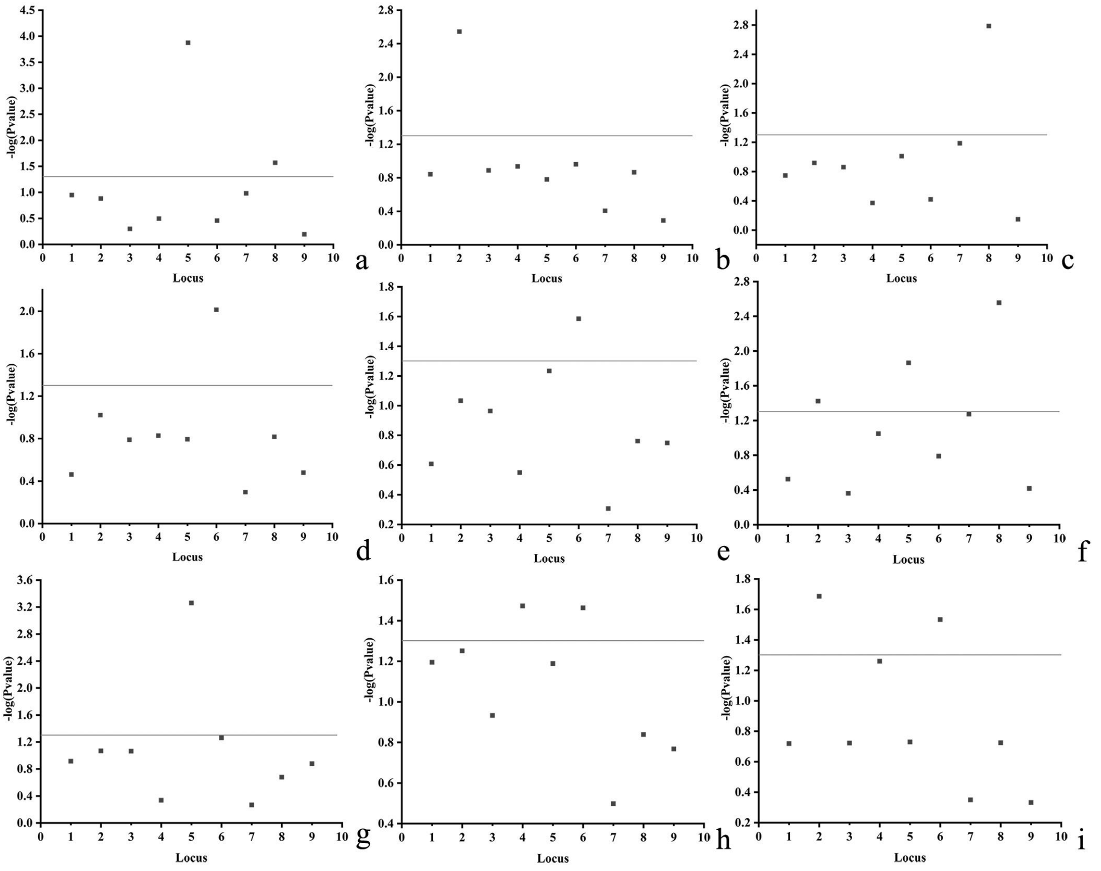

Fig. S2 Correlation analysis of phenotypic characteristics and molecular markers among populations

a. Plant height; b. Leaf length; c. Flower color; d. Amount of flowers; e. Number of branches; f. Flower diameter; g. Ground diameter; h. Ratio of leaf length to width; i. Leaf width

Above horizontal line indicates $P < 0.05$, significant correlation
